# BDNF and Schizophrenia: From Neurodevelopment to Neuronal Plasticity, Learning, and Memory

**DOI:** 10.3389/fpsyt.2013.00045

**Published:** 2013-06-17

**Authors:** R. Nieto, M. Kukuljan, H. Silva

**Affiliations:** ^1^Biomedical Neuroscience Institute, Universidad de Chile, Santiago, Chile; ^2^Clínica Psiquiátrica Universitaria, Hospital Clínico Universidad de Chile, Santiago, Chile; ^3^Laboratorio de Neurobiología Celular y Molecular, Facultad de Medicina, Universidad de Chile, Santiago, Chile

**Keywords:** Brain-derived neurotrophic factor, schizophrenia, neurodevelopment, neurotoxicity, neuroplasticity, learning, memory, cognition

## Abstract

Brain-Derived Neurotrophic Factor (BDNF) is a neurotrophin that has been related not only to neurodevelopment and neuroprotection, but also to synapse regulation, learning, and memory. Research focused on the neurobiology of schizophrenia has emphasized the relevance of neurodevelopmental and neurotoxicity-related elements in the pathogenesis of this disease. Research focused on the clinical features of schizophrenia in the past decades has emphasized the relevance of cognitive deficits of this illness, considered a core manifestation and an important predictor for functional outcome. Variations in neurotrophins such as BDNF may have a role as part of the molecular mechanisms underlying these processes, from the neurodevelopmental alterations to the molecular mechanisms of cognitive dysfunction in schizophrenia patients.

## Introduction

The brain-derived neurotrophic factor (BDNF) is the most widely distributed neurotrophin in the brain, and it has been associated with several psychiatric disorders. Evidence increasingly suggests that schizophrenia is a subtle disorder of brain development and plasticity. Considering the role BDNF in neurodevelopment, synapse regulation, and synaptic plasticity, it has been proposed as a candidate to explain part of the pathogenesis of this disease.

Alterations in neurotrophic factors such as BDNF at the protein and gene level may contribute to altered brain development, synaptic disconnectivity, and failures in neuroplasticity, and explain at least in part some of the morphological and neurochemical abnormalities found in the brains of patients with schizophrenia.

This article reviews different elements that provide evidence that BDNF may play a role in pathogenesis of schizophrenia, but also reviews the evidence suggesting that it may play a role in the pathophysiology of cognitive deficits, which can be considered core symptoms of this disease.

## BDNF Role as a Neurotrophin

### Neurotrophins in neurodevelopment and neuroprotection

During the development of the central nervous system, neural connections are established and redefined through a series of development programs involving specification of axons and dendrites, growth processes, innervation of target cells, cell death, and synaptogenesis. Many of these developmental processes are regulated by proteins derived from the target cells, which signal in a retrograde manner through long distances from the distal axons to the neuronal bodies. Neurons depend for their survival on these interactions with target cells, which release specific proteins that play a crucial role in these interactions (Leibrock et al., 1989; Zweifel et al., 2005). These specific proteins are called neurotrophins, a family of proteins that include the brain-derived neurotrophic factor (BDNF).

The first neurotrophin to be discovered was NGF, about 50 years ago. Several studies demonstrated its involvement in the regulation of neuronal death, both in response to subsequent damage to the nervous system as well as during development (Perez-Polo et al., 1990). The dependence of many neurons on their target cells for normal development and the restricted neuronal specificity of NGF (mainly peripheral nervous system), suggested the existence of other neurotrophic factors.

The structure of BDNF was reported in 1990, a neurotrophin that promotes the survival of neuronal populations located in the central nervous system, or neuronal populations directly connected to it (Barde, 1990). In the following years other neurotrophins with similar functions were identified, such as NT-3 (Hohn et al., 1990; Maisonpierre et al., 1990) and NT-6 (Gotz et al., 1994).

Neurotrophins are all small, basic, secretory proteins that allow the survival of specific neuronal populations. In its biologically active forms together show about 50% amino acid identity. The genes encoding neurotrophins are expressed not only during development but also in the adult, in a variety of tissues including the central nervous system.

Each neurotrophin can signal through two different cell surface receptors: the Trk tyrosine kinase receptor and the p75 neurotrophin receptor. Each Trk receptor is preferentially activated by one or more neurotrophin: TrkA by NGF, TrkB by BDNF, and TrkC by NT-3. Although the long-term trophic effects of neurotrophins depend on gene regulation, the cytoplasmic effectors activated by neurotrophins also exert a wide range of more rapid actions, including morphogenetic and chemotropic effects on developing neurons, and modulation of neuronal excitability and synaptic transmission (Poo, 2001; Chao, 2003).

### BDNF as synaptic modulator

Once the role of the neurotrophins as regulatory factors of neuronal survival and differentiation was properly described, new evidence indicated that they also acted as synaptic modulators. Synaptic activity regulates the synthesis, secretion, and action of neurotrophins. These, in turn, induce changes in the morphology and synaptic efficacy. Thus, the neurotrophins are involved in activity-dependent synaptic plasticity, and with long-term changes in the synaptic connections at a structural and a functional level (Poo, 2001).

Neurotrophins seem to be necessary for the maintenance of functional synapses, because their removal produces a reduction in synaptic connectivity between cultured neurons. In the central nervous system neurons, BDNF can increase the number of excitatory and inhibitory synapses, regulating axonal morphology, or directly promoting synapse formation. Additionally, BDNF promote the maturation and stabilization of the cellular and molecular components of neurotransmitters release, and this subsequently leads to an increase in the number of functional synapses. Therefore, neurotrophins regulate synapse maturation at the morphological, molecular, and functional levels. This role of BDNF in synapses is crucial not only during development but also for synaptic plasticity in the adult (Vicario-Abejón et al., 2002).

In addition to their classic effects on neuronal cell survival, neurotrophins can also regulate axonal and dendritic growth and guidance, synaptic structure and connections, neurotransmitter release, long-term potentiation, and synaptic plasticity. Neurotrophin receptors may act as a point of convergence that might be involved in the integration of many environmental inputs. This can lead to alterations in neuronal circuitry and, ultimately, in behavior. Neurotrophins can produce long-term changes in the functionality of adult neurons through changes in transcription (Chao, 2003).

Activity-dependent synaptic plasticity models, whether in the context of development or in the context of learning and memory, have long postulated the existence of extracellular signaling molecules which reinforce and stabilize the active synapses. The work of many laboratories has shown that neurotrophins such as BDNF met the two major criteria to be considered a mediator of this type: the production is regulated by neural activity, and neurotrophic factors themselves have potent effects on signaling properties of target neurons (Lo, 1995).

### BDNF in neuronal plasticity, learning, and memory

In relation to the cellular and molecular biology underlying cognitive functioning, several studies support the conclusion that BDNF plays a key role, given its participation in the processes of synaptic plasticity and learning. An increased expression of BDNF can have a positive effect on the generation of LTP and memory (Lee and Silva, 2009).

The role of BDNF in learning and memory was first demonstrated in studies at the cellular and molecular level. It has been shown that BDNF secretion is required for long-term potentiation (LTP) and long-term depression (LTD), which are the cellular mechanisms underlying learning and memory (Poo, 2001; Aicardi et al., 2004). In relation to long-term memory, is noteworthy that BDNF is sufficient to induce the transformation of early phase LTP to late phase LTP, even in the presence of protein synthesis inhibitors (Lu et al., 2005).

In animal models, the role of BDNF in cognition has also been shown in studies with BDNF mutant mice that show learning deficits and hippocampal-dependent altered pattern discrimination (Korte et al., 1996; Gorski et al., 2003). Inhibition of the signaling of BDNF also alters long-term memory in animal models (Lu et al., 2005).

## Neurobiology of Schizophrenia (Relevant to Neurotrophic Factors)

### Schizophrenia: Role of neurodevelopment and neurodegeneration

Primary alterations in the activity of neurotrophins could lead to inappropriate alterations in cortical circuitry and synaptic transmission in the developing brain, which could then translate into the neural dysfunction underlying psychiatric disorders such as schizophrenia (Favalli et al., 2012).

Evidence increasingly suggests that schizophrenia is a subtle disorder of brain development (Ross et al., 2006). Considering the neurodevelopmental hypothesis about schizophrenia, BDNF has been proposed as a candidate to explain part of the pathogenesis of this disease.

An inadequate neurotrophic support during brain development could lead to a structural disorganization of networks in which neural connections are established in a sub-optimal manner. Therefore, alterations in BDNF may contribute to altered brain development, failures in neuroplasticity, and synaptic disconnectivity, and explain at least in part some of the morphological, neurochemical, and cytoarchitectonic abnormalities found in the brains of patients with schizophrenia (Durany and Thome, 2004; Shoval and Weizman, 2005; Buckley et al., 2007a).

Subsequently, an inadequate neurotrophic support in the adult may be an underlying mechanism for a reduced brain’s ability to make adaptive changes and increased vulnerability to neurotoxic damage (Angelucci et al., 2005).

Long duration of untreated psychosis has been associated with poorer prognosis in patients with schizophrenia. Such a relationship of untreated psychosis to outcome may indicate a neurodegenerative process and may have important implications for understanding the pathophysiology of schizophrenia. Rizos et al. (2010) findings indicate that low serum BDNF levels at the onset of schizophrenia were associated with a long duration of untreated psychosis, and this could be related to an acute neurodegenerative reaction during the untreated phase of psychosis.

Ho et al. (2007) studied the relation between BDNF val66met polymorphism and progressive brain volume changes in schizophrenia. Significant genotype effects were observed on within-subject changes in volumes of frontal lobe gray matter, lateral ventricles, and sulcal CSF. Met allele carriers had significantly greater reductions in frontal gray matter volume, with reciprocal volume increases in the lateral ventricles and sulcal CSF than Val homozygous patients. Therefore, BDNF variants may be among several factors affecting progressive brain volume changes in schizophrenia.

### Synapse deregulation in schizophrenia

Over the last decades, convergent findings from various areas of investigation have suggested that alterations of synaptic transmission and neuronal connectivity might be the core feature of schizophrenia (Frankle et al., 2003). There is evidence for altered neurotransmission at glutamatergic, GABAergic, dopaminergic, cholinergic, and possibly serotonergic synapses in schizophrenia. Susceptibility genes that have a role in neural development and in synaptic plasticity may contribute to pathogenic mechanisms of schizophrenia in these synaptic pathways (Ross et al., 2006; Keshavan et al., 2008; Yin et al., 2012).

Considering BDNF functions described above, it is reasonable to think that an abnormal BDNF signaling can influence neuronal differentiation and synaptic function, leading to altered brain functioning in the synaptic systems implicated in schizophrenia (Palomino et al., 2006). Actually, BDNF has a proven role in neuronal survival and plasticity of dopaminergic neurons, cholinergic and serotonergic neurons, and all of these are pathways involved in the pathophysiology of schizophrenia (Angelucci et al., 2005; Buckley et al., 2007a).

Although the neurochemical origins of schizophrenia do not necessarily lie in dopamine dysregulation, this operates as the final common pathway underlying positive psychotic symptoms and may also play a role in negative and cognitive symptoms. In a plausible model schizophrenia can be seen as the consequence of the actions of a number of component causes, such as genes or early environmental hazards that subtly alter subsequent neurodevelopment, thereby predisposing the subject to later dopamine dysregulation (Di Forti et al., 2007).

Antipsychotic drugs act by blocking the dopamine transmission at the dopamine D2-like receptors. BDNF controls the expression of one of these D2-like receptors, the dopamine D3 receptor. This raises the hypothesis of a link between cortical area, via BDNF, and the dopamine neurotransmission pathway in schizophrenia and its treatment (Guillin et al., 2007).

The neurodevelopmental hypothesis and the dopamine hypothesis theories are beginning to be integrated through the growing evidence that all the developmental risk factors which increase risk of schizophrenia appear to act by facilitating dopamine dysregulation (Murray et al., 2008).

Early models for the etiology of schizophrenia focused on dopamine neurotransmission because of the powerful antipsychotic action of dopamine antagonists. Nevertheless, recent evidence increasingly supports a primarily glutamatergic dysfunction in this condition, where dopaminergic disbalance is a secondary effect. A current model for the pathophysiology of schizophrenia involves a dysfunctional mechanism by which a NMDA receptor hypofunction leads to a dysregulation of GABA fast-spiking interneurons, consequently disinhibiting pyramidal glutamatergic output, and disturbing the signal-to-noise ratio. This mechanism might explain better than other models some cognitive deficits observed in this disease, as well as the dopaminergic alterations and therapeutic effect of antipsychotics (Gaspar et al., 2009).

In patients with schizophrenia, a deficiency in BDNF signaling mediated by the receptor TrkB may result in a reduction of the synthesis of GABA in the dorsolateral prefrontal cortex. This can lead to an alteration in perisomatic inhibition of pyramidal neurons, contributing to a decreased capacity for frequency synchronized gamma neuronal activity, which is required for working memory (Lewis et al., 2005).

### Cognitive dysfunction in schizophrenia

#### Studies in schizophrenia patients

Cognitive deficits are a core manifestation of schizophrenia, and cognitive dysfunction has been identified as a major determinant of the long-term quality of life. The difficulties of patients with schizophrenia to live independently and to gain a competitive employment is largely due to cognitive dysfunction. Cognitive impairment has emerged as an important new target in schizophrenia therapeutics in light of evidence that cognitive deficits are critically related to the functional of disability that is characteristic of the illness (Sharma, 2003; Gold, 2004).

The level and pattern of cognitive deficits in schizophrenia has been documented using comprehensive neuropsychological batteries in dozens of studies over the past decades. There is a characteristic pattern of cognitive deficits that occur with very high frequency, the deficits are relatively stable over time, and cognitive deficits are relatively independent of the symptomatic manifestations of the illness. Thus, cognitive impairment appears to be a well-defined, reliable, and distinct dimension of the illness (Gold, 2004).

Virtually all areas of cognition in schizophrenia are altered to some degree. Measurements of working memory, episodic memory, and ideational fluency complex care issues seem to be most affected by the disease. The measures of semantic knowledge and visuoperceptual skills seem to be the least impacted. Cognitive deficits are present in other types of psychotic disorders, but are more severe and widespread in schizophrenia (Zanelli et al., 2010).

In one of the first cross-sectional studies that characterized this deficit, Bilder et al. (2000) conducted an extensive battery of neuropsychological assessments to 94 patients with first-episode schizophrenia after initial management of their clinical psychotic and a control group of 36 healthy volunteers. Patients showed a large generalized neuropsychological deficit (1.5 standard deviations compared to healthy volunteers). Additionally, this generalized deficit patients had subtle relative deficits (less than 0.5 standard deviations compared to their own average profile) in assessments of memory and executive functions. The learning and memory dysfunction was the best distinguished patients from healthy individuals followed by motor deficits. Patients with better neurocognitive performance had only memory deficits, while patients with lower cognitive performance were affected in both memory deficits and executive functions. Deficits in executive functions and attention were most related to difficulties in global functioning and worse prognosis (Bilder et al., 2000).

According to Sponheim et al. (2010), research has only recently begun to determine when cognitive dysfunction develops in individuals with schizophrenia. The available evidence provides evidence to support the existence of significant cognitive deficits prior to disease onset, but there is less information about the course of cognitive dysfunction from the onset and the chronic phase of schizophrenia. Although longitudinal studies would be optimal to determine the stability of cognitive deficits, treatment effects are often confounding factors and there is no studies based on large and representative samples of patients who have been followed for a long time. These authors evaluated the cross-sectional 41 patients with recent-onset schizophrenia and 106 patients with chronic schizophrenia, suggesting that some measures of problem solving and fine motor skills could decrease chronic patients beyond what would be expected as a result of aging. However, the findings indicate that the cognitive impairment is similar in patients with recent-onset schizophrenia and in patients with chronic schizophrenia.

Jahshan et al. (2010) followed in a period of 6 months 48 patients considered at risk of developing schizophrenia and 20 patients in their first episode of this disease. At-risk patients showed an improvement in the overall assessment of intelligence. In contrast, patients after a first-episode showed improved verbal learning but a deterioration in the working memory and processing speed. According to the authors, these inconsistent trajectories suggest that some cognitive domains may benefit from stabilization in the early phases of clinical psychotic, while others may worsen with disease progression.

Despite the central role of the cognitive manifestations of schizophrenia, it is important to note that there are some schizophrenic patients who have a standard neuropsychological performance. Estimates of the number of patients with this diagnosis showing no alteration in neurocognitive tests varies considerably between different studies, but it has been estimated that in the range between 16 and 45% of patients with schizophrenia (Reichenberg et al., 2009).

Social cognition is described as the higher mental processes that are engaged while people store, process, and use social information to make sense of themselves and others. Aspects of social cognition include emotion perception, social cue interpretation, attribution style, and theory of mind, all of which appear disordered in schizophrenia (Kucharska-Pietura and Mortimer, 2013). According to a recent meta-analysis by Savla et al. (2012), the evidence for deficits across multiple social cognitive domains (theory of mind, social perception, social knowledge, attributional bias, emotion perception, and emotion processing) in schizophrenia is clear. Emotion identification deficits are associated with functional impairments in schizophrenia (Irani et al., 2012).

One of the limitations of studies evaluating neurocognitive function in patients with schizophrenia is the wide variety of tests used. Some initiatives to determine consensus batteries to evaluate cognition have been developed in recent years, such as CNTRICS and MATRICS. The CNTRICS initiative aims to systematically address barriers to translating paradigms developed in the basic neuroscience field for use in translational research (Carter et al., 2008). To support the development of pharmacological agents to improve neurocognitive deficits of schizophrenia, NIMH launched an initiative called “Measurement and Treatment Research to Improve Cognition in Schizophrenia” (MATRICS). As a result this initiative developed a consensus neurocognitive battery for use in clinical trials to study the pharmacological effects on neurocognition (Green and Nuechterlein, 2004).

#### Studies in animal models of schizophrenia

While such initiatives developed a consensus on the core cognitive deficits of schizophrenic patients and recommended a standardized test battery to evaluate them, more recently work has begun to identify specific rodent behavioral tasks with translational relevance to specific cognitive domains affected in schizophrenia. Developing reliable, predictive animal models for complex psychiatric disorders, such as schizophrenia, is essential to increase our understanding of the neurobiological basis of the disorder and for the development of novel drugs with improved therapeutic efficacy (Jones et al., 2011).

All available animal models of schizophrenia fit into four different induction categories: developmental, drug-induced, lesion, or genetic manipulation. Most rodent models have behavioral phenotype changes that resemble “positive-like” symptoms of schizophrenia, probably reflecting altered mesolimbic dopamine function, but fewer models also show altered social interaction, and learning and memory impairment, analogous to negative, and cognitive symptoms of schizophrenia respectively (Jones et al., 2011).

Considering drug-induced animal models of schizophrenia, since phencyclidine (PCP) induces several symptoms in humans similar to those seen in schizophrenia, it has been used to attempt to produce a pharmacological rodent model of schizophrenia. Morphological changes in PCP rats including decreased hippocampal volume and neuronal number, and decreased synaptophysin mRNA all support the suggestion of synaptic dysfunction (Jones et al., 2011). Neonatal PCP also produces a sustained elevation in hippocampal and entorhinal BDNF in 8-week-old rats, similar to clinical observations in the corticolimbic system of patients with chronic schizophrenia (Takahashi et al., 2006).

In one of the developmental animal models of schizophrenia, treatment of pregnant rat dams with methylazoxymethanol acetate (MAM), an anti-mitotic and anti-proliferative agent that methylates DNA) does not affect litter size or pup body weight, but selectively affects brain development. The behavioral alterations seen in MAM offspring vary according to the gestational day (GD) of MAM administration in a sequential manner: at GD17 changes similar as those seen in schizophrenia have been observed, with impaired acquisition of learning at GD17 or later. The observation of disorganization, sporadic density, and heterotopias within the pyramidal CA3 region of the hippocampus with GD17 administration, which has also be observed in schizophrenia patients, lends weight to the use of GD17 MAM exposure as a preclinical model for schizophrenia (Jones et al., 2011). Interestingly, GD17 MAM offspring show reduced nerve growth factor (NGF) and brain-derived nerve growth factor (BDNF) in the parietal cortex at adulthood, but whether this is a cause or consequence of the neurodevelopmental changes observed is unclear (Fiore et al., 2004).

A model that simulates many of the cognitive and sensory deficits of the schizophrenia disorder is the use of random variable prenatal stress (PS) in the rat. To study both hippocampal gene expression changes in offspring of prenatally stressed dams and to address genetic variability, Neeley et al. (2011a) used a random array of prenatal stressors in three different rat strains with diverse responses to stress: Fischer, Sprague–Dawley, and Lewis rats. Expression of BDNF mRNA in the hippocampus was increased after an acute stress in all controls of each strain, yet a decrease was seen after acute stress in the PS Sprague–Dawley and Lewis rats. In both Sprague–Dawley and Lewis male rats, PS also decreased the amount of available proBDNF and mature BDNF protein in the hippocampus, and changed the activation of TrkB and its downstream signaling cascade. This reduction in BDNF the hippocampus can be related to the learning and memory deficits observed in these animal models. However, PS in the male Fischer rats did not elicit these changes, suggesting that the Fischer genetic background encodes a developmental environment that is protective during prenatal stress. Differential processing of BDNF after prenatal stress in the three rat strains has implications for human subjects, where genetic differences may protect or exacerbate the effects of an environmental stressor during fetal development (Neeley et al., 2011b).

Using mutant mice [TrkB-PV(−/−)] in which the gene for the BDNF receptor, tyrosine kinase B receptor (trkB), has been specifically deleted in parvalbumin-expressing, fast-spiking GABAergic (PV+) interneurons, Zheng et al. (2011) showed that TrkB is structurally and functionally important for the integrity of the hippocampal network. The amplitude of glutamatergic inputs to PV+ interneurons and the frequency of GABAergic inputs to excitatory pyramidal cells were reduced in the TrkB-PV(−/−) mice. Functionally, rhythmic network activity in the gamma-frequency band (30–80 Hz) was significantly decreased in hippocampal area CA1. This decrease was caused by a desynchronization and overall reduction in frequency of action potentials generated in PV+ interneurons of TrkB-PV(−/−) mice. These results show that the integration of PV+ interneurons into the hippocampal microcircuit is impaired in TrkB-PV(−/−) mice, resulting in decreased rhythmic network activity in the gamma-frequency band. These findings highlight the role of BDNF and its receptor in a crucial aspect of the neuronal circuitry of schizophrenia that is particularly related to alterations in cognitive functioning.

## BDNF Genetic Variations

### BDNF polymorphisms and schizophrenia

Several studies have looked for association of this gene and schizophrenia, with different results. One of the first authors to report an association between functional polymorphisms of BDNF (Val66Met and C-207T) and schizophrenia were Kunugi et al. (2004) and Hong et al. (2003), in different populations of Asian origin. However, other studies did not find this association, including studies in similar populations (Chen et al., 2006; Tochigi et al., 2006; Li et al., 2007). At least two meta-analyzes have ruled out this association in Asian populations, such as the study of Naoe et al. (2007) and a publication by Kawashima et al. (2009), that included results of Japanese population and a meta-analysis with other studies from Asian populations.

In Caucasian population, some of the positive results that can be mentioned include the study of Szekeres et al. (2003) for C-207T and study Skibinska et al. (2004) for Val66Met. However, in this population some studies have also failed in their attempt to prove an association for both Val66Met (Golimbert et al., 2008) and for C-270T (Galderisi et al., 2005), (Szczepankiewicz et al., 2005).

Jonsson et al. (2006) conducted a study in Swedish population and found no significant difference between cases and controls for different BDNF polymorphisms. However, they analyzed their data in combination with results from other studies and conducted a meta-analysis, in which they found that both the T allele of BDNF 270 and the homozygous state for Val66Met are associated with the presence of schizophrenia. This meta-analysis selected mainly studies in Caucasian population, including U.S. subjects (Egan et al., 2003), Finland (Anttila et al., 2004), Poland (Skibinska et al., 2004), Scotland (Neves-Pereira et al., 2002), the Netherlands (de Krom et al., 2005), Germany (Schumacher et al., 2005), and France (Gourion et al., 2005).

In general, it is thought that some variants of the polymorphism may lead to memory impairment and susceptibility to neuropsychiatric disorders (Bath and Lee, 2006). A 2007 meta-analysis of case-control studies found a relationship between BDNF Val66Met SNP and substance-related disorders, eating disorders, and schizophrenia (Gratacos et al., 2011). However, another 2007 meta-analysis could not find any association between this SNP and schizophrenia or bipolar disorder (Kanazawa et al., 2007).

Schizotypal personality traits have been associated with schizophrenia in several studies. Regarding BDNF and schizotypal traits, Ma et al. (2007) found a weak association between Val66Met and measurement of schizotypal personality questionnaire (SPQ). However, Tochigi et al. (2006) found no significant association.

The pharmacogenetic relationship between the BDNF gene and response to antipsychotic treatment with risperidone has been studied by Xu et al. (2010). Individual and combinatorial genetic variants in the BDNF gene might have a role in the therapeutic response to risperidone in the Han Chinese population.

There is a relationship between genetic variations of BDNF gene, such as Val66Met polymorphism, and BDNF serum levels. Ozan et al. (2010) demonstrated that met allele carriers are associated with lower levels of serum BDNF (23.08 vs. 26.87, *P* ≤ 0.002).

### BDNF polymorphisms and cognitive function

In the adult human brain, the site of higher expression of BDNF is the hippocampal formation (Barde, 1990). Studies on human subjects indicate that BDNF genetic alterations, such as the Val66Met SNP, are related to deficits in cognitive functions, particularly in the hippocampus-dependent memory (Egan et al., 2003; Dempster et al., 2005).

Healthy volunteers who carry the Met allele have impaired fMRI hippocampal activity when performing a declarative memory task (Hariri et al., 2003), and reduced gray matter volumes at hippocampal and prefrontal levels (Pezawas et al., 2004; Szeszko et al., 2005).

In a review of the molecular genetics of cognition, Savitz et al. (2006) concluded that it is likely that val66met BDNF variant exerts an effect on memory performance and executive function, considering studies that have demonstrated that the met allele is associated with both lower hippocampal volume or functional activity and weaker performance on tests of memory and executive function.

The BDNF Val66Met polymorphism has also been associated with emotion processing. Mukherjee et al. (2011), studied the effect of genetic variation in the BDNF gene on a common attribute such as fear processing. They used functional magnetic resonance imaging to examine the effect of the BDNF Val66Met genotype on neural activity for fear processing. Forty healthy participants performed an implicit fear task during scanning, where subjects made gender judgments from facial images with neutral or fearful emotion. Subjects were tested for facial emotion recognition post-scan. Functional connectivity was investigated using psycho-physiological interactions. Subjects were genotyped for the BDNF Val66Met polymorphism and the measures compared between genotype groups. Met carriers showed overactivation in the anterior cingulate cortex (ACC), brainstem, and insula bilaterally for fear processing, along with reduced functional connectivity from the ACC to the left hippocampus, and impaired fear recognition ability. According to the authors, these results show that during fear processing, Met allele carriers show an increased neural response in regions previously implicated in mediating autonomic arousal. Further, the Met carriers showed decreased functional connectivity with the hippocampus, which may reflect differential retrieval of emotional associations. Together, these effects show significant differences in the neural substrate for fear processing with genetic variation in BDNF.

Although limited by a small sample, a recent study by Outhred et al. (2012) contributes novel and preliminary findings relating to a functional epistasis of the 5-HTTLPR and BDNF Val66Met genes in emotion processing, and provides guidance on appropriate methods to determine genetic epistasis in fMRI.

### BDNF polymorphisms and cognitive function in schizophrenia

Rybakowski et al. (2006) investigated the relationship between Val66Met polymorphisms of the BDNF gene and prefrontal cognitive function in 129 patients with schizophrenia. Cognitive tests included the Wisconsin Card Sorting Test (WCST), with such domains as number of perseverative errors, non-perseverative errors, completed corrected categories, conceptual level responses, and set to the first category, and the N-back test, where mean reaction time and percent of correct reactions were measured. No relationship between Val66Met polymorphism of the BDNF gene and the results of the WCST was observed, but patients with Val/Val genotype had a higher percentage of correct reactions in the N-back test than those with the remaining genotypes.

More recently, Lu et al. (2012) found that schizophrenic patients with Met allele showed more percent WCST perseverative errors than those without Met allele. After stratification based on gender, an association between the Met allele and a higher percentage of perseverative errors was found in male patients, but not in females. According to these authors, the effect of Val66Met polymorphism involvement in impaired executive function may have gender-specific characteristics.

Zhang et al. (2012a) evaluated a group of schizophrenic inpatients with the repeatable battery for the assessment of neuropsychological status (RBANS), and assessed the presence of the BDNF Val66Met polymorphism and serum BDNF levels. They found an association between the BDNF Met variant and poor visuospatial/constructional performance and attentional decrements in schizophrenic patients. They also found that the association between decreased BDNF serum levels and cognitive impairment in schizophrenia is dependent on the BDNF Val66Met polymorphism.

Eisenberg et al. (2013) studied 47 medication-free patients with schizophrenia or schizoaffective disorder and 74 healthy comparison individuals with genotyping for the Val(66)Met SNP and [(15)O]H(2)O positron emission tomography (PET) to measure resting and working memory-related hippocampal regional cerebral blood flow (rCBF). In patients, harboring a Met allele was associated with significantly less hippocampal rCBF. This finding was opposite to the genotype effect seen in healthy participants, resulting in a significant diagnosis-by-genotype interaction. Exploratory analyses of interregional resting rCBF covariation revealed a specific and significant diagnosis-by-genotype interaction effect on hippocampal-prefrontal coupling. A diagnosis-by-genotype interaction was also found for working memory-related hippocampal rCBF change, which was uniquely attenuated in Met allele-carrying patients. Thus, both task-independent and task-dependent hippocampal neurophysiology accommodates a Met allelic background differently in patients with schizophrenia than in control subjects.

## BDNF Levels in the Brain and Peripheral Blood

### BDNF levels and schizophrenia

Some studies have focused in brain BDNF levels in postmortem studies of schizophrenic patients. Durany et al. (2001) found a significant increase in BDNF concentrations in cortical areas and a significant decrease of this neurotrophin in hippocampus of patients when compared to control. Takahashi et al. (2000) found that levels of brain-derived neurotrophic factor (BDNF) were elevated specifically in the anterior cingulate cortex and hippocampus of schizophrenic patients, and that the expression of TrkB receptor were reduced significantly in the hippocampus or the prefrontal cortex. Nevertheless, Weickert et al. (2003) detected a significant reduction in BDNF mRNA and protein in the DLPFC of patients with schizophrenia compared to normal individuals.

These findings are important to lend further evidence for the understanding of the neurotrophin hypothesis of schizophrenic psychoses. However, this approach lack the possibility to have a dynamic study of changes in BDNF levels in relation to the schizophrenic process and its clinical evolution.

A valid approach for studying the role of BDNF in schizophrenia patients is the measurement of plasma or serum levels. BDNF’s ability to cross the blood-brain barrier suggests that BDNF levels measured in peripheral blood may reflect levels in the brain (Pan et al., 1998). The relation between peripheral and brain BDNF has been demonstrated by a recent study that showed parallel changes in BDNF levels in plasma and CSF in patients with schizophrenia, indicating that plasma BDNF levels reflect the brain changes in BDNF levels (Pillai et al., 2010).

Reports of BDNF plasma levels in patients with chronic schizophrenia in comparison to control subjects have been somewhat inconsistent. Despite most studies describe lower levels in patients (Toyooka et al., 2002; Tan et al., 2005; Grillo et al., 2007), some even found higher levels in patients(Jockers-Scherubl et al., 2004).

In studies with treatment-naive patients who had their first psychotic episode, Buckley et al. (2007b) found a significant reduction in plasma levels of BDNF (135 ± 21.77 pg/ml) compared with control subjects (290.5 ± 38.8 pg/ml). In a similar population, Rizos et al. (2008) also found BDNF levels in serum significantly lower in patients with first-episode schizophrenia (23.92 ± 5.99 ng/ml) compared with control subjects (30, 0 ± 8.43). However, a previous study comparing the cross section of BDNF levels in antipsychotic-naive patients, medicated patients, and control subjects, with no differences between these groups (Shimizu et al., 2003).

Chen da et al. (2009) also found significantly lower BDNF levels in first-episode patients with schizophrenia in comparison to healthy control subjects (9.0 ± 4.2 ng/ml vs. 12.1 ± 2.2 ng/ml), and observed a significant positive correlation between BDNF levels and PANSS positive subscore. Furthermore, higher BDNF levels were observed in patients with paranoid subtype of schizophrenia. They concluded that low BDNF levels at the onset of psychosis suggest that it may contribute to the pathogenesis of schizophrenia and perhaps, could be a candidate biological marker for positive symptoms.

Palomino et al. (2006) was one of the first authors to study prospectively the evolution of BDNF plasma levels of patients with a final diagnosis of schizophrenia was measured at baseline at the start of their first psychotic episode (4.19 ± 2.26 ng/ml) and the following months after starting treatment (often with atypical antipsychotics), with a higher level at 6 months duration (6.53 ± 2.48 ng/ml), approaching the level of the control subjects (7.55 ± 4.31 ng/ml).

Many authors have also evaluated the effects of both pharmacological and non-pharmacological treatments for SCZ in BDNF, and despite some controversial results, it seems that both medicated and non-medicated patients present with lower levels of BDNF when compared to controls. Further data suggests that typical antipsychotics may decrease BDNF expression whereas mixed results have been obtained with atypical antipsychotics (Favalli et al., 2012).

In a recent review, Martinotti et al. (2012) suggested that most data points to decreased plasma and serum BDNF concentrations in both drug-naive and medicated schizophrenic patients compared to healthy controls. They also mentioned that there are contrasting results on a possible correlation between increase in BDNF concentrations and treatment with antipsychotics.

In a meta-analysis, Green et al. (2011) concluded that blood levels of BDNF are reduced in medicated and drug-naive patients with schizophrenia. This evidence is of moderate quality, that is, precise but with considerable, unexplained heterogeneity across study results.

The relationship between BDNF plasma levels and treatment response to antipsychotic treatment with risperidone has been studied by Lee and Kim (2009). They found that baseline BDNF levels were significantly lower in non-response patients than others, suggesting that higher plasma BDNF levels might be associated with better response to antipsychotic treatment.

### BDNF levels and cognitive function

Considering that hippocampal volume shrinks in late adulthood, and that circulating levels of BDNF decline with advancing age, Erickson et al. (2010) tested whether age-related reductions in serum levels of BDNF would be related to shrinkage of the hippocampus and memory deficits in older adults. Hippocampal volume was acquired by automated segmentation of magnetic resonance images in 142 older adults without dementia. The caudate nucleus was also segmented and examined in relation to levels of serum BDNF. Spatial memory was tested using a paradigm in which memory load was parametrically increased. The results showed that increasing age was associated with smaller hippocampal volumes, reduced levels of serum BDNF, and poorer memory performance.

Interestingly, lower levels of BDNF were associated with smaller hippocampi and poorer memory, even when controlling for the variation related to age. In an exploratory mediation analysis, hippocampal volume mediated the age-related decline in spatial memory and BDNF mediated the age-related decline in hippocampal volume. Caudate nucleus volume was unrelated to BDNF levels or spatial memory performance. These results identify serum BDNF as a significant factor related to hippocampal shrinkage and memory decline in late adulthood (Erickson et al., 2010).

However, Niitsu et al. (2011) reported that serum BDNF levels of normal controls showed negative correlations with verbal working memory.

### BDNF levels and cognitive deficits in schizophrenia

Rizos et al. (2011) investigated the correlation between serum BDNF levels and hippocampal volumes in a sample of first-episode drug-naïve patients with schizophrenia and healthy control subjects and found that both hippocampal volume and serum BDNF levels were decreased in these patients. A significant positive association was found between serum BDNF and the corrected right hippocampal volume in the group of patients, such that the smaller the hippocampal volume, the more reduced the serum BDNF levels.

These findings indicate that low serum BDNF levels are associated with reduction in hippocampal volume at the onset of schizophrenia and may further support the theory of a neuroprogressive-neurotoxic reaction associated with the onset of psychosis (Rizos et al., 2011).

Recently, Zhang et al. (2012a) evaluated a group of schizophrenic inpatients with the repeatable battery for the assessment of neuropsychological status (RBANS), and assessed the presence of the BDNF Val66Met polymorphism and serum BDNF levels. They found an association between the BDNF Met variant and poor visuospatial/constructional performance and attentional decrements in schizophrenic patients. They also found that the association between decreased BDNF serum levels and cognitive impairment in schizophrenia is dependent on the BDNF Val66Met polymorphism.

In a different publication, Zhang et al. (2012b) also reported that BDNF serum levels are positively associated specifically with immediate memory in schizophrenia.

Niitsu et al. (2011) reported that serum BDNF levels of schizophrenic patients showed positive correlations with the scores of the Scale for the Assessment of Negative Symptoms (SANS) and the Information subtest scores of Wechsler Adult Intelligence Scale Revised (WAIS-R).

In a study by de Azua et al. (2013) in first-episode psychosis patients, there was a positive association between plasma BDNF and five cognitive domains (learning ability, immediate and delayed memory, abstract thinking and processing speed) after a 6 month follow-up. The association persisted after controlling for medications prescribed, drug use, intelligence quotient (IQ), and negative symptoms. In the healthy control group, BDNF levels were not associated with cognitive test scores.

Carlino et al. (2011) hypothesized that controversial data about serum BDNF in relation to cognition in schizophrenia and control subjects might be due to the differential regulation of BDNF precursor pro-BDNF and proteolytic products mature and truncated-BDNF. Their results suggest that deficiency in pro-BDNF processing might be a possible biological mechanism underlying schizophrenia with cognitive impairment.

According to Pedrini et al. (2011), serum BDNF levels concentration in schizophrenia merits further investigations with regard to the role of neurotrophins in the cognitive response to treatment with atypical antipsychotics.

Intensive cognitive training can improve cognitive processes relevant to psychosocial functioning in schizophrenia. One example of this is the neuroplasticity-based auditory training used to improve verbal memory (Fisher et al., 2009). In patients with schizophrenia receiving neurocognitive training, Vinogradov et al. (2009) found a significant increase in plasma levels of BDNF during the period of cognitive remediation therapy. In this context, BDNF has also been proposed as a biomarker of cognitive enhancement.

## Conclusion

Brain-Derived Neurotrophic Factor (BDNF) is a neurotrophin that has been related not only to neurodevelopment and neuroprotection, but also to synapse regulation, learning, and memory.

In relation to the pathogenesis of schizophrenia, particularly neurodevelopmental and neurotoxicity-related elements, neurotrophins such as BDNF can provide an explanatory framework supported by evidence at molecular, cellular, animal, and human subjects levels. In addition, the synaptic alterations due to problems in BDNF expression may alter neurotransmitter pathways classically involved in schizophrenia pathophysiology, such as the dopaminergic and GABA systems.

The cognitive deficits of this illness, considered a core manifestation and an important predictor for functional outcome, can be understood in the context of the molecular and cellular mechanisms of learning and memory, in which BDNF plays a key role through regulation of synaptic plasticity. This is related to the findings of behavioral studies in animal models and the results of studies of cognition in human subjects.

Since BDNF is widely distributed in the central nervous system and has been implicated in several psychiatric illnesses, the dysregulation of BDNF signaling is not specific to schizophrenia. However, considering its relation to key core mechanisms in the etiology and pathophysiology of schizophrenia, it can potentially become an important biomarker of the clinical status and/or the prognosis of patients with this disease.

A better knowledge of BDNF signaling in schizophrenic patients and controls may have important implications for the treatment of schizophrenia, considering current trends toward individualized medicine based in molecular biomarkers at gene and protein levels.

In conclusion, evidence support the notion that variations in neurotrophins such as BDNF may have a role in the molecular mechanisms underlying this disease, from the neurodevelopmental alterations to the molecular mechanisms of cognitive dysfunction in patients with schizophrenia.

## Conflict of Interest Statement

The authors declare that the research was conducted in the absence of any commercial or financial relationships that could be construed as a potential conflict of interest.

## References

[B1] Aicardi G. Argilli E. Capello S. Santi S. Riccio M. Thoenen H. (2004). Induction of long-term potentiation and depression is reflected by corresponding changes in secretion of endogenous brain-derived neurotrophic factor. Proc. Natl. Acad. Sci. U.S.A. 101, 15788–15792 10.1073/pnas.0406960101 15505222 PMC524856

[B2] Angelucci F. Brene S. Mathé A. A. (2005). BDNF in schizophrenia, depression and corresponding animal models. Mol. Psychiatry 10, 345–352 10.1038/sj.mp.4001637 15655562

[B3] Anttila S. Illi A. Kampman O. Mattila K. M. Lehtimaki T. Leinonen E. (2004). Lack of association between two polymorphisms of brain-derived neurotrophic factor and response to typical neuroleptics. J. Neural Transm. 112, 885–890 10.1007/s00702-004-0233-9 15526143

[B4] Barde Y. A. (1990). The nerve growth factor family. Prog. Growth Factor Res. 2, 237–248 10.1016/0955-2235(90)90021-B 2133291

[B5] Bath K. Lee F. (2006). Variant BDNF (Val66Met) impact on brain structure and function. Cogn. Affect. Behav. Neurosci. 6, 79–85 16869232 10.3758/cabn.6.1.79

[B6] Bilder R. M. Goldman R. S. Robinson D. Reiter G. Bell L. Bates J. A. (2000). Neuropsychology of first-episode schizophrenia: initial characterization and clinical correlates. Am. J. Psychiatry 157, 549–559 10.1176/appi.ajp.157.4.549 10739413

[B7] Buckley P. Mahadik S. Pillai A. Terry A. (2007a). Neurotrophins and schizophrenia. Schizophr. Res. 94, 1–11 10.1016/j.schres.2007.01.025 17524622

[B8] Buckley P. F. Pillai A. Evans D. Stirewalt E. Mahadik S. (2007b). Brain derived neurotrophic factor in first- episode psychosis. Schizophr. Res. 91, 1–5 10.1016/j.schres.2006.12.026 17306505 PMC1933504

[B9] Carlino D. Leone E. Di Cola F. Baj G. Marin R. Dinelli G. (2011). Low serum truncated-BDNF isoform correlates with higher cognitive impairment in schizophrenia. J. Psychiatr. Res. 45, 273–279 10.1016/j.jpsychires.2010.06.012 20630543

[B10] Carter C. S. Barch D. M. Buchanan R. W. Bullmore E. Krystal J. H. Cohen J. (2008). Identifying cognitive mechanisms targeted for treatment development in schizophrenia: an overview of the first meeting of the Cognitive Neuroscience Treatment Research to Improve Cognition in Schizophrenia Initiative. Biol. Psychiatry 64, 4–10 10.1016/j.biopsych.2008.03.020 18466880 PMC2577821

[B11] Chao M. (2003). Neurotrophins and their receptors: a convergence point for many signaling pathways. Nat. Rev. Neurosci. 4, 299–309 10.1038/nrn1078 12671646

[B12] Chen Q. Y. Chen Q. Feng G. Y. Wan C. L. Lindpaintner K. Wang L. J. (2006). Association between the brain-derived neurotrophic factor (BDNF) gene and Schizophrenia in the Chinese population. Neurosci. Lett. 397, 285–290 16406671 10.1016/j.neulet.2005.12.033

[B13] Chen da C. Wang J. Wang B. Yang S. C. Zhang C. X. Zheng Y. L. (2009). Decreased levels of serum brain-derived neurotrophic factor in drug-naïve first-episode schizophrenia: relationship to clinical phenotypes. Psychopharmacology (Berl.) 207, 375–380 10.1007/s00213-009-1665-6 19787338

[B14] de Azua S. Matute C. Stertz L. Mosquera F. Palomino A. De la Rosa I. (2013). Plasma brain-derived neurotrophic factor levels learning capacity and cognition in patients with first episode psychosis. BMC Psychiatry 13:27 10.1186/1471-244X-13-27 23320462 PMC3567944

[B15] de Krom M. Bakker S. C. Hendriks J. van Elburg A. Hoogendoorn M. Verduijn W. (2005). Polymorphisms in the brain-derived neurotrophic factor gene are not associated with either anorexia nervosa or schizophrenia in Dutch patients. Psychiatr. Genet. 15, 81 10.1097/00041444-200506000-00003 15900221

[B16] Dempster E. Toulopoulou T. McDonald C. Bramon E. Walshe M. Fibley F. (2005). Association between BDNF val66met genotype and episodic memory. Am. J. Med. Genet. B Neuropsychiatr. Genet. 134, 73–75 15719396 10.1002/ajmg.b.30150

[B17] Di Forti M. Lappin J. Murray R. (2007). Risk factors for schizophrenia – all roads lead to dopamine. Eur. Neuropsychopharmacol. 17(Suppl. 2), S101–S107 10.1016/j.euroneuro.2007.02.005 17336764

[B18] Durany N. Michel T. Zöchling R. Boissl K. W. Cruz-Sánchez F. F. Riederer P. (2001). Brain-derived neurotrophic factor and neurotrophin 3 in schizophrenic psychoses. Schizophr. Res. 52, 79–86 10.1016/S0920-9964(00)00084-0 11595394

[B19] Durany N. Thome J. (2004). Neurotrophic factors and the pathophysiology of schizophrenic psychoses. Eur. Psychiatry 19, 326–337 10.1016/j.eurpsy.2004.06.020 15363470

[B20] Egan M. F. Kojima M. Callicot J. H. Goldberg T. E. Kolachana B. S. Bertolino A. (2003). The BDNF val66met polymorphism affects activity-dependent secretion of BDNF and human memory and hippocampal function. Cell 112, 257–269 10.1016/S0092-8674(03)00035-7 12553913

[B21] Eisenberg D. P. Ianni A. M. Wei S. M. Kohn P. D. Kolachana B. Apud J. (2013). Brain-derived neurotrophic factor (BDNF) Val(66)Met polymorphism differentially predicts hippocampal function in medication-free patients with schizophrenia. Mol. Psychiatry 18, 713–720 10.1038/mp.2012.187 23319002 PMC3628926

[B22] Erickson K. I. Prakash R. S. Voss M. W. Chaddock L. Heo S. McLaren M. (2010). Brain-derived neurotrophic factor is associated with age-related decline in hippocampal volume. J. Neurosci. 30, 5368–5375 10.1523/JNEUROSCI.6251-09.2010 20392958 PMC3069644

[B23] Favalli G. Li J. Belmonte-de-Abreu P. Wong A. H. Daskalakis Z. J. (2012). The role of BDNF in the pathophysiology and treatment of schizophrenia. J. Psychiatr. Res. 46, 1–11 10.1016/j.jpsychires.2011.09.022 22030467

[B24] Fiore M. Grace A. A. Korf J. Stampachiacchiere B. Aloe L. (2004). Impaired brain development in the rat following prenatal exposure to methylazoxymethanol acetate at gestational day 17 and neurotrophin distribution. Neuroreport 15, 1791–1795 10.1097/01.wnr.0000135934.03635.6a 15257149

[B25] Fisher M. Holland C. Merzenich M. M. Vinogradov S. (2009). Using neuroplasticity-based auditory training to improve verbal memory in schizophrenia. Am. J. Psychiatry 166, 805–811 10.1176/appi.ajp.2009.08050757 19448187 PMC2720319

[B26] Frankle G. Lerma J. Laurelle M. (2003). The synaptic hypothesis of schizophrenia. Neuron 39, 205–216 10.1016/S0896-6273(03)00423-9 12873379

[B27] Galderisi S. Maj M. Kirkpatrick B. Piccardi P. Mucci A. Invernizzi G. (2005). COMT Val(158)Met and BDNF C(270)T polymorphisms in schizophrenia: a case-control study. Schizophr. Res. 73, 27–30 10.1016/j.schres.2004.06.017 15567073

[B28] Gaspar P. A. Bustamante L. Silva H. Aboitiz A. (2009). Molecular mechanisms underlying glutamatergic dysfunction in schizophrenia: therapeutic implications. J. Neurochem. 111, 891–900 10.1111/j.1471-4159 19686383

[B29] Gold J. M. (2004). Cognitive deficits as treatment targets in schizophrenia. Schizophr. Res. 72, 21–28 10.1016/j.schres.2004.09.008 15531404

[B30] Golimbert V. E. Korovaitseva G. I. Abramova L. I. Kasparov S. V. Uvarova L. G. (2008). Association of the Val66Met polymorphism of the brain-derived neurotrophic factor gene with schizophrenia in Russians. Mol. Biol. 42, 531–535 10.1134/S0026893308040079 18856059

[B31] Gorski J. A. Balogh S. A. Wehner J. M. Jones K. R. (2003). Learning deficits in forebrain-restricted brain-derived neurotrophic factor mutant mice. Neuroscience 121, 341–354 10.1016/S0306-4522(03)00426-3 14521993

[B32] Gotz R. Koster R. Winkler C. Raulf F. Lottspeich F. Scharti M. (1994). Neurotrophin-6 is a new member of the nerve growth factor family. Nature 372, 266–269 10.1038/372266a0 7969471

[B33] Gourion D. Goldberger C. Leroy S. Bourdel M. C. Olié J. P. Krebs M. O. (2005). Age at onset of schizophrenia: interaction between brain-derived neurotrophic factor and dopamine D3 receptor gene variants. Neuroreport 16, 1407–1410 10.1097/01.wnr.0000175245.58708.6b 16056149

[B34] Gratacos M. González J. R. Mercader J. de Cid R. Urretavizcaya M. Estivill X. (2011). Brain-derived neurotrophic factor Val66Met and psychiatric disorders: meta-analysis of case-control studies confirm association to substance-related disorders, eating disorders, and schizophrenia. Biol. Psychiatry 61, 911–912 10.1016/j.biopsych.2006.08.025 17217930

[B35] Green M. F. Nuechterlein K. (2004). The MATRICS initiative: developing a consensus cognitive battery for clinical trials. Schizophr. Res. 72, 1–3 10.1016/j.schres.2004.09.006 15531401

[B36] Green M. J. Matheson S. L. Shepherd A. Weickert C. S. Carr V. J. (2011). Brain-derived neurotrophic factor levels in schizophrenia: a systematic review with meta analysis. Mol. Psychiatry 16, 960–972 10.1038/mp.2010.88 20733577

[B37] Grillo R. W. Ottoni G. L. Leke R. Souza D. O. Portela L. V. Lara D. R. (2007). Reduced serum BDNF levels in schizophrenic patients on clozapine or typical antipsychotics. J. Psychiatr. Res. 41, 31–35 10.1016/j.jpsychires.2006.01.005 16546213

[B38] Guillin O. Demily C. Thibaut F. (2007). Brain-derived neurotrophic factor in schizophrenia and its relation with dopamine. Int. Rev. Neurobiol. 78, 377–395 10.1016/S0074-7742(06)78012-6 17349867

[B39] Hariri A. R. Goldberg T. E. Mattay V. S. Kolachana B. S. Callicott J. H. Egan M. F. (2003). Brain-derived neurotrophic factor val66met polymorphism affects human memory-related hippocampal activity and predicts memory performance. J. Neurosci. 23, 6690–6694 12890761 10.1523/JNEUROSCI.23-17-06690.2003 PMC6740735

[B40] Ho B. C. Andreasen N. Dawson J. Wassink T. (2007). Association between brain-derived neurotrophic factor Val66Met gene polymorphism and progressive brain volume changes in schizophrenia. Am. J. Psychiatry 164, 1890–1899 10.1176/appi.ajp.2007.05111903 18056245 PMC3062255

[B41] Hohn A. Leibrock J. Bailey K. Barde Y. A. (1990). Identification and characterization of a novel member of the nerve growth factor/brain-derived neurotrophic factor familiy. Nature 344, 229–241 10.1038/344339a0 2314473

[B42] Hong C. J. Yu Y. W. Lin C. H. Tsai S. J. (2003). An association study of a brain-derived neurotrophic factor Val66Met polymorphism and clozapine response of schizophrenic patients. Neurosci. Lett. 349, 206–208 10.1016/S0304-3940(03)00828-0 12951204

[B43] Irani F. Seligman S. Kamath V. Kohler C. Gur R. C. (2012). A meta-analysis of emotion perception and functional outcomes in schizophrenia. Schizophr. Res. 137, 203–211 10.1016/j.schres.2012.01.023 22341200 PMC3351501

[B44] Jahshan C. Heaton R. K. Golshan S. Cadenhead K. S. (2010). Course of neurocognitive deficits in the prodrome and first episode of schizophrenia. Neuropsychology 24, 109–120 10.1037/a0016791 20063952 PMC2808194

[B45] Jockers-Scherubl M. C. Danker-Hopfe H. Mahlberg R. Selig F. Rentzsch J. Schurer F. (2004). Brain-derived neurotrophic factor serum concentrations are increased in drug-naïve schizophrenic patients with chronic cannabis abuse and multiple substance abuse. Neurosci. Lett. 371, 79–83 10.1016/j.neulet.2004.08.045 15500971

[B46] Jones C. A. Watson D. J. Fone K. C. (2011). Animal models of schizophrenia. Br. J. Pharmacol. 164, 1162–1194 10.1111/j.1476-5381.2011.01386.x 21449915 PMC3229756

[B47] Jonsson E. Edman-Ahlbom B. Sillen A. Gunnar A. Kulle B. Frigessi A. (2006). Brain-derived neurotrophic factor gene (BDNF) variants and schizophrenia: an association study. Prog. Neuropsychopharmacol. Biol. Psychiatry 30, 924–933 10.1016/j.pnpbp.2006.02.008 16581172

[B48] Kanazawa T. Glatt S. J. Kia-Keating B. Yoneda H. Tsuang M. T. (2007). Meta-analysis reveals no association of the Val66Met polymorphism of brain-derived neurotrophic factor with either schizophrenia or bipolar disorder. Psychiatr. Genet. 17, 165–170 10.1097/YPG.0b013e32801da2e2 17417060

[B49] Kawashima J. Ikeda M. Kishi T. Kitakima T. Yamanouchi Y. Kinoshita Y. (2009). BDNF is not associated with schizophrenia: data from a Japanese population study and meta-analysis. Schizophr. Res. 112, 72–79 10.1016/j.schres.2009.03.040 19406621

[B50] Keshavan M. Tandon R. Boutros N. Nasrallah H. (2008). Schizophrenia, “just the facts”: what we know in 2008. Part 3: neurobiology. Schizophr. Res. 106, 89–107 10.1016/j.schres.2008.07.020 18799287

[B51] Korte M. Griesbeck O. Gravel C. Carroll P. Staiger V. Thoenen H. (1996). Virus-mediated gene transfer into hippocampal CA1 region restores long-term potentiation in brain-derived neurotrophic factor mutant mice. Proc. Natl. Acad. Sci. U.S.A. 93, 12547–12552 10.1073/pnas.93.22.12547 8901619 PMC38029

[B52] Kucharska-Pietura K. Mortimer A. (2013). Can antipsychotics improve social cognition in patients with schizophrenia? CNS Drugs 27, 335–343 10.1007/s40263-013-0047-0 23533009 PMC3657085

[B53] Kunugi H. Iijima Y. Tatsumi M. Yoshida M. Hashimoto R. Kato T. (2004). No association between the Val66Met polymorphism of the brain-derived neurotrophic factor gene and bipolar disorder in a Japanese population: a multicenter study. Biol. Psychiatry 56, 376–378 10.1016/j.biopsych.2004.06.017 15336520

[B54] Lee B. H. Kim Y. K. (2009). Increased plasma brain-derived neurotrophic factor, not nerve growth factor-beta, in schizophrenia patients with better response to risperidone treatment. Neuropsychobiology 59, 51–58 19270464 10.1159/000205518

[B55] Lee Y. Silva A. (2009). The molecular and cellular biology of enhanced cognition. Nat. Rev. Neurosci. 10, 126–139 10.1038/nrn2572 19153576 PMC2664745

[B56] Leibrock J. Lottspeich F. Hohn A. Hofer M. Hengerer B. Masiakowski P. (1989). Molecular cloning and expression of brain-derived neurotrophic factor. Nature 341, 149–152 10.1038/341149a0 2779653

[B57] Lewis D. A. Hashimoto T. Volk D. W. (2005). Cortical inhibitory neurons and schizophrenia. Nat. Rev. Neurosci. 6, 312–324 10.1038/nrn1648 15803162

[B58] Li W. Wei J. Zhou D. F. Tan Y. L. Cao Y. L. Zhang X. Y. (2007). Lack of association between the BDNF C270T polymorphism and schizophrenia in a Chinese Han population. Schizophr. Res. 97, 297–298 17669628 10.1016/j.schres.2007.07.005

[B59] Lo D. (1995). Neurotrophic factors and synaptic plasticity. Neuron 15, 978–981 10.1016/0896-6273(95)90085-3 7576664

[B60] Lu B. Pang P. Woo N. (2005). The yin and yang of neurotrophin action. Nat. Rev. Neurosci. 6, 603–614 10.1038/nrn1726 16062169

[B61] Lu W. Zhang C. Yi Z. Li Z. Wu Z. Fang Y. (2012). Association between BDNF Val66Met polymorphism and cognitive performance in antipsychotic-naïve patients with schizophrenia. J. Mol. Neurosci. 47, 505–510 10.1007/s12031-012-9750-4 22477643

[B62] Ma X. Sun J. Yao J. Wang Q. Hu X. Deng W. (2007). A quantitative association study between schizotypal traits and COMT, PRODH and BDNF genes in a healthy Chinese population. Psychiatry Res. 153, 7–15 10.1016/j.psychres.2007.02.003 17604122

[B63] Maisonpierre P. C. Belluscio L. Squinto S. Ip N. Y. Furth M. E. Lindsay R. M. (1990). Neurotrophin 3: a neurotrophic factor related to NGF and BDNF. Science 247, 1446–1451 10.1126/science.2321006 2321006

[B64] Martinotti G. Di Iorio G. Marini S. Ricci V. De Berardis D. Di Giannantonio M. (2012). Nerve growth factor and brain-derived neurotrophic factor concentrations in schizophrenia: a review. J. Biol. Regul. Homeost. Agents 26, 347–356 23034254

[B65] Mukherjee P. Whalley H. C. McKirdy J. W. McIntosh A. M. Johnstone E. C. Lawrie S. M. (2011). Effects of the BDNF Val66Met polymorphism on neural responses to facial emotion. Psychiatry Res. 191, 182–188 10.1016/j.pscychresns.2010.10.001 21310593

[B66] Murray R. M. Lappin J. Di Forti M. (2008). Schizophrenia: from developmental deviance to dopamine dysregulation. Eur. Neuropsychopharmacol. 18(Suppl. 3), S129–34 10.1016/j.euroneuro.2008.04.002 18499406

[B67] Naoe Y. Shinkai T. Hori H. Fukunaka Y. Utsunomiya K. Sakata S. (2007). No association between the brain derived neurotrophic factor Val66Met polymorphism and schizophrenia in Asian populations: evidence from a case-control study and meta-analysis. Neurosci. Lett. 415, 108–112 10.1016/j.neulet.2007.01.006 17267117

[B68] Neeley E. Berger R. Koening J. Leonard S. (2011a). Strain dependent effects of prenatal stress on gene expression in the rat hippocampus. Physiol. Behav. 104, 334–339 10.1016/j.physbeh.2011.02.032 21382392 PMC3118943

[B69] Neeley E. Berger R. Koenig J. I. Leonard S. (2011b). Prenatal stress differentially alters brain-derived neurotrophic factor expression and signaling across rat strains. Neuroscience 187, 24–35 10.1016/j.neuroscience.2011.03.065 21497180 PMC3114039

[B70] Neves-Pereira M. Mundo E. Muglia P. King N. Macciardi F. Kennedy J. L. (2002). The brain-derived neurotrophic factor gene confers susceptibility to bipolar disorder: evidence from a family-based association study. Am. J. Hum. Genet. 71, 651–655 10.1086/342288 12161822 PMC379201

[B71] Niitsu T. Shirayama Y. Matsuzawa D. Hasegawa T. Kanahara N. Hashimoto T. (2011). Associations of serum brain-derived neurotrophic factor with cognitive impairments and negative symptoms in schizophrenia. Prog. Neuropsychopharmacol. Biol. Psychiatry 35, 1836–1840 10.1016/j.pnpbp.2011.09.004 21930178

[B72] Outhred T. Das P. Dobson-Stone C. Griffiths K. Felmingham K. L. Bryant R. A. (2012). The functional epistasis of 5-HTTLPR and BDNF Val66Met on emotion processing: a preliminary study. Brain Behav. 2, 778–788 10.1002/brb3.99 23170240 PMC3500464

[B73] Ozan E. Okur H. Eker C. Eker O. Gonul A. Akarsu N. (2010). The effect of depression, BDNF gene val66met polymorphism and gender on serum BDNF levels. Brain Res. Bull. 81, 61–65 10.1016/j.brainresbull.2009.06.022 19589373

[B74] Palomino A. Vallejo-Illarramendi A. González-Pinto A. Aldama A. González-Gómez C. Mosquera F. (2006). Decreased levels of plasma BDNF in first-episode schizophrenia and bipolar disorder patients. Schizophr. Res. 86, 321–322 10.1016/j.schres.2006.05.028 16829047

[B75] Pan W. Banks W. A. Fasold M. B. Bluth J. Kastin A. J. (1998). Transport of brain-derived neurotrophic factor across the blood-brain barrier. Neuropharmacology 37, 1553–1561 10.1016/S0028-3908(98)00141-5 9886678

[B76] Pedrini M. Chendo I. Grande I. Lobato M. I. Belmonte-de-Abreu P. S. Lersch C. (2011). Serum brain-derived neurotrophic factor and clozapine daily dose in patients with schizophrenia: a positive correlation. Neurosci. Lett. 491, 207–210 10.1016/j.neulet.2011.01.039 21256922

[B77] Perez-Polo J. R. Foreman P. J. Jackson G. R. Shan D. Taglialatela G. Thorpe L. W. (1990). Nerve growth factor and neuronal cell death. Mol. Neurobiol. 4, 57–91 10.1007/BF02935585 2076219

[B78] Pezawas L. Verchinski B. A. Mattay V. S. Callicott J. H. Kolachana B. S. Strayb R. E. (2004). The brain-derived neurotrophic factor val66met polymorphism and variation in human cortical morphology. J. Neurosci. 24, 4401–4411 10.1523/JNEUROSCI.2680-04.2004 15537879 PMC6730170

[B79] Pillai A. Kale A. Joshi S. Naphade N. Raju M. S. Nasrallah H. (2010). Decreased BDNF levels in CSF of drug-naive first-episode psychotic subjects: correlation with plasma BDNF and psychopathology. Int. J. Neuropsychopharmacol. 13, 535–539 10.1017/S1461145709991015 19941699

[B80] Poo M. (2001). Neurotrophins as synaptic modulators. Nat. Rev. Neurosci. 2, 24–32 10.1038/35049004 11253356

[B81] Reichenberg A. Harvey P. D. Bowie C. R. Mojtabai R. Rabinowitz J. Heaton R. K. (2009). Neuropsychological function and dysfunction in schizophrenia and psychotic affective disorders. Schizophr. Bull. 35, 1022–1029 10.1093/schbul/sbn044 18495643 PMC2728814

[B82] Rizos E. Rontos I. Laskos E. Arsenis G. Michalopoulou P. G. VAsilopoulos D. (2008). Investigation of serum BDNF levels in drug-naïve patients with schizophrenia. Prog. Neuropsychopharmacol. Biol. Psychiatry 32, 1308–1311 10.1016/j.pnpbp.2008.04.007 18502013

[B83] Rizos E. N. Michalopoulou P. G. Siafakas N. Stefanis N. Douzenis A. Rontos I. (2010). Association of serum brain-derived neurotrophic factor and duration of untreated psychosis in first-episode patients with schizophrenia. Neuropsychobiology 62, 87–90 10.1159/000315438 20523079

[B84] Rizos E. N. Papathanasiou M. Michalopoulou P. G. Mazioti A. Douzenis A. Kastania A. (2011). Association of serum BDNF levels with hippocampal volumes in first psychotic episode drug-naive schizophrenic patients. Schizophr. Res. 129, 201–204 10.1016/j.schres.2011.03.011 21470828

[B85] Ross C. Margolis R. Reading S. Plenikov M. Coyle J. (2006). Neurobiology of schizophrenia. Neuron 52, 139–115 10.1016/j.neuron.2006.09.015 17015232

[B86] Rybakowski J. K. Borkowska A. Skibinska M. Szczepankiewicz A. Kapelski P. Leszczynska-Rodziewicz A. (2006). Prefrontal cognition in schizophrenia and bipolar illness in relation to Val66Met polymorphism of the brain-derived neurotrophic factor gene. Psychiatry Clin. Neurosci. 60, 70–76 10.1111/j.1440-1819.2006.01462.x 16472361

[B87] Savitz J. Solms M. Ramesar R. (2006). The molecular genetics of cognition: dopamine, COMT and BDNF. Genes Brain Behav. 5, 311–328 10.1111/j.1601-183X.2005.00163.x 16716201

[B88] Savla G. N. Vella L. Armstrong C. C. Penn D. L. Twamley E. W. (2012). Deficits in domains of social cognition in schizophrenia: a meta-analysis of the empirical evidence. Schizophr. Bull. [Epub ahead of print]. 10.1093/schbul/sbs080 22949733 PMC3756768

[B89] Schumacher J. Abou Jamra R. Becker T. Ohlraun S. Klopp N. Binder E. B. (2005). Evidence for a relationship between genetic variants at the brain-derived neurotrophic factor (BDNF) locus and major depression. Biol. Psychiatry 58, 307–314 10.1016/j.biopsych.2005.04.006 16005437

[B90] Sharma T. (2003). Characterisation of cognitive impairment in schizophrenia. Lancet Neurol. 2, 10 10.1016/S1474-4422(03)00257-6 12849291

[B91] Shimizu E. Hashimoto K. Watanabe H. Komatsu N. Okamura N. Koike K. (2003). Serum brain-derived factor (BDNF) levels in schizophrenia are indistinguishable from controls. Neurosci. Lett. 351, 111–114 10.1016/j.neulet.2003.08.004 14583394

[B92] Shoval H. Weizman A. (2005). The possible role of neurotrophins in the pathogenesis and therapy of schizophrenia. Eur. Neuropsychopharmacol. 15, 319–329 10.1016/j.euroneuro.2004.12.005 15820422

[B93] Skibinska M. Hauser J. Czerski P. Leszczynska-Rodziewicz A. Kosmowska M. Kapelski P. (2004). Association analysis of brain-derived neurotrophic factor (BDNF) gene Val66Met polymorphism in schizophrenia and bipolar affective disorder. World J. Biol. Psychiatry 5, 215–220 10.1080/15622970410029936 15543516

[B94] Sponheim S. R. Jung R. E. Seidman L. J. Mesholam-Gately R. I. Manoach D. S. O’Leary D. S. (2010). Cognitive deficits in recent-onset and chronic schizophrenia. J. Psychiatr. Res. 44, 421–428 10.1016/j.jpsychires.2009.09.010 19878956 PMC3940967

[B95] Szczepankiewicz A. Skibinska M. Czerski P. M. Kapelski P. Leszczynska-Rodziewicz A. Słopien A. (2005). No association of the brain-derived neurotrophic factor (BDNF) gene C-270T polymorphism with schizophrenia. Schizophr. Res. 76, 187–193 15949651 10.1016/j.schres.2005.02.006

[B96] Szekeres G. Juhasz A. Rimanoczy A. Keri S. Kanka Z. (2003). The C270T polymorphism of the brain-derived neurotrophic factor gene is associated with schizophrenia. Schizophr. Res. 65, 15–18 10.1016/S0920-9964(02)00505-4 14623369

[B97] Szeszko P. R. Lipsky R. Mentschel C. Robinson D. Gunduz-Bruce H. Sevy S. (2005). Brain-derived neurotrophic factor val66met polymorphism and volume of the hippocampal formation. Mol. Psychiatry 10, 631–636 10.1038/sj.mp.4001656 15768049

[B98] Takahashi M. Kakita A. Futamura T. Watanabe Y. Mizuno M. Sakimura K. (2006). Sustained brain-derived neurotrophic factor up-regulation and sensorimotor gating abnormality induced by postnatal exposure to phencyclidine: comparison with adult treatment. J. Neurochem. 99, 770–780 10.1111/j.1471-4159.2006.04106.x 16903871

[B99] Takahashi M. Shirakawa O. Toyooka K. Kitamura N. Hashimoto T. Maeda K. (2000). Abnormal expression of brain-derived neurotrophic factor and its receptor in the corticolimbic system of schizophrenic patients. Mol. Psychiatry 5, 293–300 10.1038/sj.mp.4000718 10889532

[B100] Tan Y. L. Zhou D. F. Cao L. Y. Zou Y. Z. Zhang X. Y. (2005). Decreased BDNF in serum of patients with chronic schizophrenia on long-term treatment with antipsychotics. Neurosci. Lett. 382, 27–32 10.1016/j.neulet.2005.02.054 15911116

[B101] Tochigi M. Otowa T. Suga M. Rogers M. Minato T. Yamasue H. (2006). No evidence for an association between the BDNF Val66Met polymorphism and schizophrenia or personality traits. Schizophr. Res. 87, 45–47 10.1016/j.schres.2006.06.029 16854566

[B102] Toyooka K. Asama K. Watanabe Y. Muratake T. Takahashi M. Someya T. (2002). Decreased levels of brain-derived neurotrophic factor in serum of chronic schizophrenic patients. Psychiatry Res. 110, 249–257 10.1016/S0165-1781(02)00127-0 12127475

[B103] Vicario-Abejón C. Owens D. McKay R. Segal M. (2002). Role of neurotrophins in central synapse formation and stabilization. Nat. Rev. Neurosci. 3, 965–974 10.1038/nrn988 12461553

[B104] Vinogradov S. Fisher M. Holland C. Shelly W. Wolkowitz O. Mellon S. H. (2009). Is serum brain-derived neurotrophic factor a biomarker for cognitive enhancement in schizophrenia? Biol. Psychiatry 66, 549–553 10.1016/j.biopsych.2009.02.017 19368899 PMC4691262

[B105] Weickert C. S. Hyde T. M. Lipska B. K. Herman M. M. Weinberger D. R. Kleinman J. E. (2003). Reduced brain-derived neurotrophic factor in prefrontal cortex of patients with schizophrenia. Mol. Psychiatry 8, 592–610 10.1038/sj.mp.4001308 12851636

[B106] Xu M. Li S. Xing Q. Gao R. Feng G. Lin Z. (2010). Genetic variants in the BDNF gene and therapeutic response to risperidone in schizophrenia patients: a pharmacogenetic study. Eur. J. Hum. Genet. 18, 707–712 10.1038/ejhg.2009.238 20087404 PMC2987331

[B107] Yin D. M. Chen Y. J. Sathyamurthy A. Xiong W. C. Mei L. (2012). Synaptic dysfunction in schizophrenia. Adv. Exp. Med. Biol. 970, 493–516 10.1007/978-3-7091-0932-8_22 22351070

[B108] Zanelli J. Reichenberg A. Morgan K. Fearon P. Kravariti E. Dazzan P. (2010). Specific and generalized neuropsychological deficits: a comparison of patients with various first-episode psychosis presentations. Am. J. Psychiatry 167, 78–85 10.1176/appi.ajp.2009.09010118 19952077

[B109] Zhang X. Y. Chenda C. Xiu M. H. Haile C. N. Luo X. Xu K. (2012a). Cognitive and serum BDNF correlates of BDNF Val66Met gene polymorphism in patients with schizophrenia and normal controls. Hum. Genet. 131, 1187–1195 10.1007/s00439-012-1150-x 22362486 PMC3671849

[B110] Zhang X. Y. Liang J. Chenda C. Xiu M. H. Yang F. D. Kosten T. A. (2012b). Low BDNF is associated with cognitive impairment in chronic patients with schizophrenia. Psychopharmacology (Berl.) 222, 277–284 10.1007/s00213-012-2643-y 22274000

[B111] Zheng K. An J. J. Yang F. Xu W. Xu Z. Q. Wu J. (2011). TrkB signaling in parvalbumin-positive interneurons is critical for gamma-band network synchronization in hippocampus. Proc. Natl. Acad. Sci. U.S.A. 108, 17201–17206 10.1073/pnas.1114241108 21949401 PMC3193255

[B112] Zweifel L. S. Kuruvilla R. Ginty D. D. (2005). Functions and mechanisms of retrograde neurotrophin signalling. Nat. Rev. Neurosci. 6, 615–625 10.1038/nrn1727 16062170

